# The Effects of Transforming Growth Factor-*β*1 on the Differentiation of Cell Organoids Composed of Gingiva-Derived Stem Cells

**DOI:** 10.1155/2022/9818299

**Published:** 2022-07-14

**Authors:** Young-Min Song, Kyung-Hwan Na, Hyun-Jin Lee, Jun-Beom Park

**Affiliations:** ^1^Department of Periodontics, College of Medicine, The Catholic University of Korea, Seoul 06591, Republic of Korea; ^2^Department of Medicine, Graduate School, The Catholic University of Korea, Seoul 06591, Republic of Korea

## Abstract

This study was aimed at evaluating the effects of transforming growth factor-*β* on the differentiation and mRNA expression of organoids made out of human mesenchymal stem cells. Cell organoids composed of gingiva-derived stem cells were cultured in the presence of transforming growth factor-*β*1 at concentrations ranging from 0, 1, 10, to 20 ng/ml. Evaluations of the cell morphology of the organoids were performed on days 7, 9, 11, and 14. Quantitative cellular viability was completed on day 14. Alkaline phosphatase activity assays were performed to evaluate the differentiation of stem cells on day 14. Real-time polymerase chain reactions were used to determine the expression levels of TGF-*β*1, RUNX2, OCN, SOX9, and COL1A1 mRNA on day 14. The stem cells produced well-formed organoids on day 7, and the addition of transforming growth factor-*β*1 did not result in relevant changes in their shape. The organoids grew in size and became more intact with longer incubation times. On day 14, the diameters were 222.2 ± 9.6, 186.1 ± 4.8, 197.2 ± 9.6, and 211.1 ± 19.2 m for transforming growth factor-*β*1 at final concentrations of 0, 1, 10, and 20 ng/ml, respectively. Quantitative cell viability results from day 14 exhibited no significant difference between the groups (*P* > 0.05). There was significantly higher alkaline phosphatase activity with the addition of transforming growth factor-*β*1 with the highest value for the 1 ng/ml group (*P* < 0.05). Real-time polymerase chain reaction results demonstrated that the mRNA expression levels of RUNX2, OCN, and SOX were higher in 1 ng/ml but did not reach statistical significance. Treatment with 1 ng/ml of transforming growth factor-*β*1 significantly increased COL1A1 mRNA expression at day 14. The application of transforming growth factor-*β*1 increased differentiation, which was confirmed by alkaline phosphatase activity and mRNA expression while maintaining cell viability.

## 1. Introduction

Recent advances have been made in three-dimensional organoids, bioengineering, and organ-on-a-chip technology for fabricating multicellular tissues [1]. One of the three-dimensional cultures of spheroids has advantages over two-dimensional cultures in that it mimics in vivo circumstances better with enhanced cell-to-cell interactions and regeneration capabilities [2]. Cells can also grow in size and can interact with their surroundings [3]. In a previous report, hybrid spheroids were fabricated through the fusion progenitor cell spheroids, endothelial cell spheroids, and supporting stem cells to achieve increased differentiation [4]. More recently, cellular self-assembly has led to the mimicry of the complexity of organ structures producing organoids including the intestine and brain [5]. Organoids can be fabricated from stem cells or progenitor cells through self-organization [6]. Organoid models have been applied for modeling human diseases, drug screening, and designing drug therapy [7]. Organoids made from human teeth demonstrated epithelial stemness phenotypes and differentiation potential with long-term expandability [8]. Organoids possess cellular structures which maintain and provide stability to the key characteristics of the targeted organs [9]. Historically, dentin-pulp-like organoids displaying stem cell-like and odontoblastic characteristics have been developed and have been tested for tooth regeneration [10]. Furthermore, organoid models for the regeneration of the periodontal ligament have been developed in vitro [11]. Bone organoids exhibit the features of microvessel formation and osteogenesis [12]. Similarly, cartilage organoids present evidence of cartilage development and maturation of the tissue [12]. Efforts have been made in organoid technology to reach similarities in matrix context and structure to native tissue [13].

Mesenchymal stem cells have been chosen as candidates in regenerative medicine because of their high proliferation and multilineage potential [2, 14]. Mesenchymal stem cells have been isolated and characterized from the waste of various dental tissues [15–19]. Various active molecules including transforming growth factor-*β*1 have been reported to be involved in tissue differentiation and repair [20]. Bone marrow-derived mesenchymal stem cells overexpressing transforming growth factor-*β*1 enhanced new bone formation and bone-related markers in animal model [21]. Transforming growth factor-*β*1 was reported to be involved in dynamic interaction with dentin sialophosphoprotein found in odontoblasts, dentin, and dental pulp [22]. Laser-activated transforming growth factor-*β*1 was reported to differentiate dental stem cells, which can be applied for regenerative purposes [23]. This study was aimed at evaluating the effects of transforming growth factor-*β*1 on the differentiation and mRNA expression of organoids made out of human mesenchymal stem cells.

## 2. Materials and Methods

### 2.1. Study Design

The Institutional Review Board of Seoul St. Mary's Hospital, College of Medicine, The Catholic University of Korea, approved the protocol of the present study after reviewing the documentation (KC21SASE0473; approval date 6 July 2021). Gingiva-derived mesenchymal stem cells (GMSCs) were obtained and characterized based on the previous publications [24]. The epithelium of the gingiva was removed from the participant. The tissue was cut in small pieces and enzymes were applied afterwards. The obtained stem cells were placed on a culture plate, and the media were replaced every two to three days.

### 2.2. Fabrication of the Stem Cell Organoids

**Figure 1:**
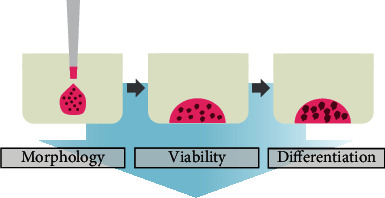
Schematic overview of the present study's design.

The dome method was performed by thawing the basement membrane matrix (phenol red-free, lactose dehydrogenase elevating virus-free, Matrigel®, Corning, NY, USA) for organoid culturing (Figure 1). The vials of the basement membrane matrix (Matrigel®) were submerged in ice. Once the basement membrane matrix (Matrigel®) was thawed, the material was pipetted up and down to ensure even dispersion. The thawed basement membrane matrix (Matrigel®) for organoid culturing was placed in a sterile culture plate that had been sprayed with 70% ethanol and air dried. The culture plate was placed in a 37°C environment overnight. A dry bath set at 37°C was used so that the temperature of the plate was consistent during the procedure.

Carefully dispensed droplets of the basement membrane matrix (Matrigel®) were applied into the middle of the wells, and the plate was set on a dry bath for at least ten minutes until the domes were polymerized. After the domes were fully polymerized, growth cell culture media were carefully added to the wells so as not to disturb the basement membrane matrix (Matrigel®) dome. The final concentrations of recombinant human transforming growth factor-*β*1 (ab50036, Abcam, Cambridge, United Kingdom) were 0, 1, 10, and 20 ng/ml. After full polymerization of the domes, the plate was placed into an incubator.

### 2.3. Harvesting of the Organoids

On days 7, 9, 11, and 14, cell culture medium from the culture plate was removed as much as possible without disturbing the cells. Prechilled cell recovery solution (Product Number 354253, Corning), which is recommended for the recovery of cells cultured on the basement membrane matrix (Matrigel®) for subsequent analyses with a volume greater than twice the basement membrane matrix (Matrigel®) volume, was added. The solution was pipetted up and down gently using wide orifice tips in order to break the basement membrane matrix (Matrigel®) without damaging the three-dimensional cultures. The cultures were then incubated with cell recovery solution (Corning) at 4°C for approximately 20 minutes.

The cultures were visualized under a microscope to determine if the basement membrane matrix (Matrigel®) had been fully depolymerized and three-dimensional cultures floating freely from the basement membrane matrix (Matrigel®). The cell recovery solution was removed and reapplied in order to repeat the process.

The cultures were briefly centrifuged to separate the structures from the solution after the three-dimensional cultures were freed from the basement membrane matrix (Matrigel®). The cell recovery solution (Corning) was then removed, and the cultures were washed with cold phosphate-buffered saline (LB 004-02, Welgene, Gyeongsan-si, Gyeongsangbuk-do, Republic of Korea) several times.

After removing the phosphate-buffered saline (Welgene), 1 ml of TRIzol reagent (TR 118, TRI Reagent®, Molecular Research Center, Inc., Cincinnati, OH, USA) was immediately added, and the solution was stored at 4°C for approximately 2 minutes and then pipetted before the samples were collected in 1.5 ml tubes and stored at -80°C.

### 2.4. Quantitative Determination of Cell Viability Using Spectrometric Analyses

Cell Counting Kit-8 (CK04-11, Dojindo, Tokyo, Japan) was used for the analysis of quantitative cell viability of the cell organoids on day 14 [25]. After adding tetrazolium and monosodium salt, the cell organoids were cultured for 60 minutes at 37°C. Absorbance was measured at 450 nm.

### 2.5. Evaluation of the Activity of Alkaline Phosphatase

The activity of alkaline phosphatase, which is based on para-nitrophenylphosphate, was used to evaluate the osteogenic differentiation of stem cell organoids on days 7, 9, 11, and 14 using commercially available kits (AS-72146, SensoLyte pNPP Alkaline Phosphatase Assay Kit, Anaspec Inc., Freemont, CA, USA) [26]. Colorimetric reaction was analyzed at room temperature for 30 minutes, and the absorbance was measured at 405 nm.

### 2.6. Total RNA Extraction and Quantification of TGF-*β*1, RUNX2, OCN, SOX9, and COL1A1 mRNA by Real-Time Quantitative Polymerase Chain Reactions (qPCRs)

Total RNA extraction was performed using commercially available kits (Thermo Fisher Scientific, Inc., Waltham, MA, USA) according to the manufacturer's instructions on day 14 [27]. mRNA expression was detected by qPCR on day 14. We used GenBank to design the sense and antisense primers for PCR assays. The primer sequences were as follows: TGF-*β*1 (accession no.: NM_000660.7, forward primer 5′-GAGCCTGAGGCCGACTACTA-3′, reverse primer 5′-AGATTTCGTTGTGGGTTTCC-3′), RUNX2 (accession no.: NM_001015051.3, forward primer 5′-CAGTTCCCAAGCATTTCATCC-3′, reverse primer 5′-AGGTGGCTGGATAGTGCATT-3′), OCN (accession no.: NM_199173.6, forward primer 5′-GGTGCAGAGTCCAGCAAAGG-3′, reverse primer 5′-GCGCCTGGGTCTCTTCACTA-3′), SOX9 (accession no.: NM_000346.4, forward primer 5′-CTGGGAACAACCCGTCTACA-3′, reverse primer 5′-GGATCATCTCGGCCATCTTC-3′), and COL1A1 (accession no.: NM_000088.4, forward primer, 5′-TACCCCACTCAGCCCAGTGT-3′, reverse primer 5′-CCGAACCAGACATGCCTCTT-3′).

### 2.7. Statistical Analysis

The data were presented as the mean of the experiments plus the standard error of the mean. A test of normality and the equality of variances in the samples were conducted. A two-way analysis of variance with post hoc analysis was used to assess the effects of concentrations and time. A one-way analysis of variance was performed with post hoc analysis for evaluation of the effects of loading concentration applying a commercially available program (SPSS 12 for Windows, SPSS Inc., Chicago, IL, USA) with a level of significance at 0.05. For each analysis, three experimental replicates were examined.

## 3. Results

### 3.1. Evaluation of Cell Morphology

**Figure 2:**
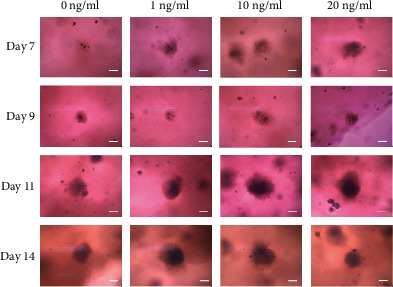
Evaluation of cell morphology on days 7, 9, 11, and 14 using inverted microscopy following treatment with different concentrations of transforming growth factor-*β* at final concentrations of 1, 10, and 20 ng/ml in osteogenic media (original magnification ×200). The bar indicates 100 *μ*m.

**Figure 3:**
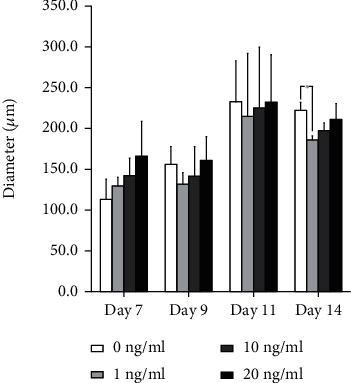
Diameter of the organoids on days 7, 9, 11, and 14. No statistically significant differences were identified with transforming growth factor-*β* application on day 7 (*P* > 0.05). Furthermore, no statistically significant differences were demonstrated in the group treated with transforming growth factor-*β* compared with longer incubation times on days 9 and 11 (*P* > 0.05).

The morphology of the organoids treated with transforming growth factor-*β*1 at final concentrations of 1, 10, and 20 ng/ml on day 7 is shown in Figure 2. The organoids grew larger and were more intact with longer incubation times. On day 7, the diameters were 113.2 ± 24.7, 129.7 ± 10.4, 142.2 ± 21.5, and 166.2 ± 42.3 *μ*m for transforming growth factor-*β*1 at final concentrations of 0, 1, 10, and 20 ng/ml, respectively (*P* > 0.05). On day 14, the diameters were 222.2 ± 9.6, 186.1 ± 4.8, 197.2 ± 9.6, and 211.1 ± 19.2 m for transforming growth factor-*β*1 at final concentrations of 0, 1, 10, and 20 ng/ml, respectively (*P* < 0.05) (Figure 3).

### 3.2. Determination of Quantitative Cellular Viability

**Figure 4:**
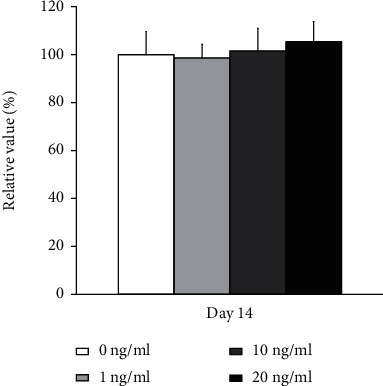
Cellular viability using the CCK-8 assay on day 14. The application did not produce significant changes in cellular viability (*P* > 0.05).

The quantitative values for cellular viability on day 14 are shown in Figure 4. The relative values for transforming growth factor-*β*1 at concentrations 1, 10, and 20 ng/ml were 98.7% ± 5.6%, 101.5% ± 9.4%, and 105.5% ± 8.3%, respectively, when the control was considered 100% (100.0% ± 9.6%) (*P* > 0.05).

### 3.3. Alkaline Phosphatase Activity Assays

**Figure 5:**
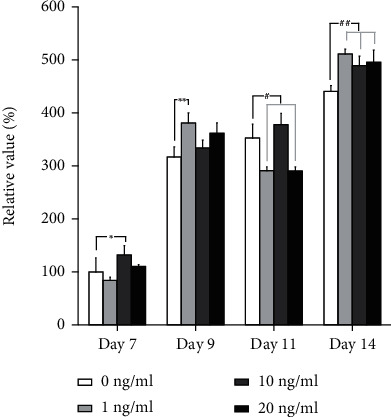
Alkaline phosphatase activity on days 7, 9, 11, and 14. ^∗^*P* < 0.05 versus the 0 nM on day 7. ^∗∗^*P* < 0.05 versus the 0 nM groups on day 9. ^#^*P* < 0.05 versus the 0 nM group on day 11. ^##^*P* < 0.05 versus the 0 nM group on day 14.

The alkaline phosphatase activity treated with transforming growth factor-*β*1 on days 7, 9, 11, and 14 is shown in Figure 5. The relative values for transforming growth factor-*β*1 at concentrations 1, 10, and 20 ng/ml on day 7 were 84.3% ± 5.7%, 132.2% ± 17.3%, and 110.6% ± 3.3%, respectively, when the control was considered 100% (100.0% ± 26.7%) (*P* < 0.05). The relative values for transforming growth factor-*β*1 at concentrations 1, 10, and 20 ng/ml on day 14 were 511.5% ± 9.0%, 489.4% ± 17.6%, and 495.7% ± 23.0%, respectively, when the control was considered 100% (440.7% ± 10.9%) (*P* < 0.05).

### 3.4. Total RNA Extraction and Quantification of TGF-*β*1, RUNX2, OCN, SOX9, and COL1A1 mRNA by qPCR

**Figure 6:**
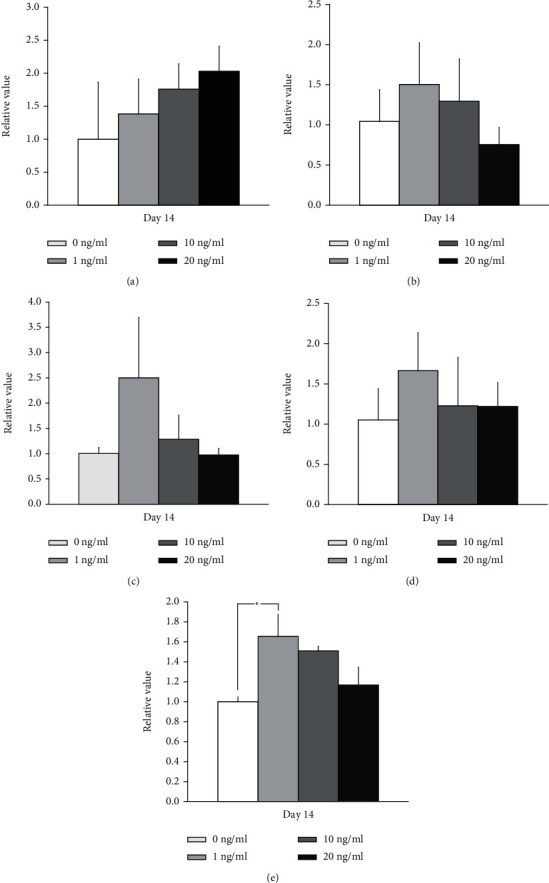
Quantification of the expression of mRNA by real-time polymerase chain reactions on day 14. (a) Quantification of the expression of TGF-*β* mRNA by real-time polymerase chain reactions on day 14. No statistically significant differences were noted when compared with the 0 ng/ml group (*P* > 0.05). (b) Quantification of the expression of RUNX2 mRNA by real-time polymerase chain reactions on day 14. No statistically significant differences were noted when compared with the 0 ng/ml group (*P* > 0.05). (c) Quantification of the expression of OCN mRNA by real-time polymerase chain reactions on day 14. No statistically significant differences were noted when compared with the 0 ng/ml group (*P* > 0.05). (d) Quantification of the expression of SOX9 mRNA by real-time polymerase chain reactions on day 14. No statistically significant differences were noted when compared with the 0 ng/ml group (*P* > 0.05). (e) Quantification of the expression of COL1A1 mRNA by real-time polymerase chain reactions on day 14. Statistically significant differences were found, when compared to the 0 ng/ml group (*P* < 0.05).

qPCR revealed that the mRNA levels of TGF-*β*1 for transforming growth factor-*β*1 at concentrations 0, 1, 10, and 20 ng/ml on day 14 were 1.001 ± 0.058, 1.384 ± 0.864, 1.761 ± 0.527, and 2.030 ± 0.382, respectively, on day 14 (*P* > 0.05) (Figure 6(a)). qPCR revealed that the mRNA levels of RUNX2 for transforming growth factor-*β*1 at concentrations 0, 1, 10, and 20 ng/ml on day 14 were 1.044 ± 0.394, 1.504 ± 0.520, 1.294 ± 0.527, and 0.755 ± 0.212, respectively, on day 14 (*P* > 0.05) (Figure 6(b)). qPCR revealed that the mRNA levels of OCN for transforming growth factor-*β*1 at concentrations 0, 1, 10, and 20 ng/ml on day 14 were 1.004 ± 0.118, 2.502 ± 1.191, 1.286 ± 0.475, and 0.974 ± 0.132, respectively, on day 14 (*P* > 0.05) (Figure 6(c)). qPCR revealed that the mRNA levels of SOX9 for transforming growth factor-*β*1 at concentrations 0, 1, 10, and 20 ng/ml on day 14 were 1.053 ± 0.387, 1.666 ± 0.470, 1.228 ± 0.603, and 1.220 ± 0.297, respectively, on day 14 (*P* > 0.05) (Figure 6(d)). qPCR revealed that the mRNA levels of COL1A1 for transforming growth factor-*β*1 at concentrations 0, 1, 10, and 20 ng/ml on day 14 were 1.001 ± 0.050, 1.656 ± 0.218, 1.509 ± 0.043, and 1.167 ± 0.177, respectively, on day 14 (*P* > 0.05) (Figure 6(e)).

## 4. Discussion

This study tested the effects of transforming growth factor-*β*1 on cellular viability and osteogenic differentiation using cell organoids made out of stem cells. The use of transforming growth factor-*β*1 increased differentiation, which was confirmed by alkaline phosphatase activity and mRNA expression while maintaining cell viability.

The important key factors for tissue engineering includes cells, growth factors, and scaffolds [28]. In a previous report, the combination of bone marrow-derived stem cells and periodontal ligament cells leading to multiphasic constructs produced superior results when compared with gingiva-derived cell sheets [29]. Gingiva-derived stem cells originate from the neural crest, and they have been actively applied in the field of dentistry for tissue regeneration [30–32]. Gingiva-derived stem cells have great advantage that they can be obtained during routine procedures under local anesthesia with easy accessibility and less morbidity [24]. Paracrine effects were higher in the three-dimensional cultures fabricated with gingiva-derived stem cells when compared with two-dimensional cultures [33]. Moreover, various agents have been applied to increase the functionality of stem cells composed of gingiva-derived stem cells with higher efficacy from the combination approach [31, 34].

To overcome bony deformities in the oral and maxillofacial region, autogenous, allogenic, or xenogenic bone grafts have been used for bone regeneration [35]. This approach can be considered as an attractive and reproducible approach [36]. However, various methods have been suggested such as cell therapy using viable cells including stem cells that have been proposed as an improved method to enhance biologic responses [37]. The advantage of the organoid model is the application of stem cells without the use of scaffolds [38]. Organoids can be fabricated from participants with healthy or diseased conditions and can mimic individual conditions [9]. Lineage-specific organoids have been fabricated and tested for cardiac regeneration [39]. Organoid models for bone regeneration are being tested, and the protocol needs to be further developed for the maturation of the organoids performed in vitro [40]. A previous report demonstrated that cartilaginous organoids made out of pluripotent stem cell-derived promoted the scaffold-free healing of long bone defects with critical size [35]. Furthermore, the development of blood vessels and communication with surrounding tissue can lead to greater development and more wide use of the organoids in bone regeneration [41]. The control of the coating material for the culturing dish and loading density of the cells may influence the characteristics of the organoids [42]. This study showed that the soundness of differentiation potential develops better with longer incubation times.

Transforming growth factor-*β* signaling has been shown to regulate osteogenic differentiation through Wnt signaling [43]. Moreover, transforming growth factor-*β* signaling is known to be involved in the differentiation of odontoblasts [44]. Transforming growth factor-*β* can be released during caries development and is reported to be involved in the repair of dentin [45]. Two receptor types for transforming growth factor-*β* have been implicated in transforming growth factor-*β*-induced signaling [46]. The expression level of transforming growth factor-*β* receptor type 1 was higher in reparative dentin when compared with normal dentin [47]. Animal models with the deletion of transforming growth factor-*β* receptor type 2 in bone-producing mesenchyme led to an alteration in osteoblast organization [48]. Similarly, the deletion of transforming growth factor-*β* receptor type 2 in odontoblastic precursor cells resulted in the abnormal development of osteodentin [49].

In this study, we fabricated organoids from a single cell type of gingiva-derived stem cells [50]. Cellular viability was tested using various methods [51, 52]. Propidium iodide was used due to the characteristics of labeling dead cells [51]. The 3-(4,5-dimethylthiazol-2-yl)-2,5-diphenyltetrazolium bromide assay evaluated the activity of mitochondrial enzymes [52]. Quantitative cell viability test was conducted using the Cell Counting Kit-8 (Dojindo, Tokyo, Japan) which is based on the use of a water-soluble tetrazolium salt-8 solution [53]. Alkaline phosphatase activity was used to evaluate the differentiation of stem cells using p-nitrophenyl phosphate as a phosphatase substrate [26]. The specific osteogenic markers (RUNX2, OCN, and COL1A1) and the chondrogenic marker (SOX9) were compared with the quantification of expression by real-time polymerase chain reactions. RUNX2 is essential for osteogenesis and osteoblast maturation and is an important regulator of the ALP, COL1A1, and OCN genes. ALP and COL1A1 are matrix-mineralizing proteins, and their expression is important for bone matrix assembly. TGF-*β*1, RUNX2, OCN, SOX9, and COL1A1 are also considered crucial for odontoblast differentiation and are related with tooth-related gene expressions [54]. Osteocalcin is considered as an odontogenic gene marker [55]. The role of the SOX9 gene in biological processes includes organogenesis and tooth development [56]. COL1A1 is associated with tooth regeneration, and the patterns of expression of COL1A1-GFP transgenes during odontoblast differentiation correlate with the expression of DSPP [57]. Although RUNX2, OCN, and COL1A1 expression did not exhibit statistically significant differences compared with the 0 ng/mL group, an enhancement of ALP activity was observed. As expected, SOX9 (a chondrogenic marker) expression was unaffected.

## 5. Conclusions

The application of transforming growth factor-*β*1 increased differentiation, which was confirmed by alkaline phosphatase activity and mRNA expression, while maintaining cell viability. Based on these findings, we concluded that transforming growth factor-*β*1 could be applied for the enhanced differentiation of cell organoids.

## Data Availability

All data analyzed during this study are included in this published article.

## References

[1] Matsumoto R., Yamamoto T., Takahashi Y. (2021). Complex organ construction from human pluripotent stem cells for biological research and disease modeling with new emerging techniques. *International Journal of Molecular Sciences*.

[2] Findeisen L., Bolte J., Vater C. (2021). Cell spheroids are as effective as single cells suspensions in the treatment of critical-sized bone defects. *BMC Musculoskeletal Disorders*.

[3] Y Baena A. R., Casasco A., Monti M. (2022). Hypes and Hopes of Stem Cell Therapies in Dentistry: a Review. *Stem Cell Reviews and Reports*.

[4] Song L., Yuan X., Jones Z. (2019). Assembly of human stem cell-derived cortical spheroids and vascular spheroids to model 3-D brain-like tissues. *Scientific Reports*.

[5] Wan A. C. A. (2016). Recapitulating cell-cell interactions for organoid construction-are biomaterials dispensable?. *Trends in Biotechnology*.

[6] Gao X., Wu Y., Liao L., Tian W. (2021). Oral organoids: progress and challenges. *Journal of Dental Research*.

[7] Shah S. B., Singh A. (2017). Cellular self-assembly and biomaterials-based organoid models of development and diseases. *Acta Biomaterialia*.

[8] Hemeryck L., Hermans F., Chappell J. (2022). Organoids from human tooth showing epithelial stemness phenotype and differentiation potential. *Cellular and Molecular Life Sciences*.

[9] Zhou Z., Cong L., Cong X. (2021). Patient-derived organoids in precision medicine: drug screening, organoid-on-a-chip and living organoid biobank. *Frontiers in Oncology*.

[10] Jeong S. Y., Lee S., Choi W. H., Jee J. H., Kim H. R., Yoo J. (2020). Fabrication of dentin-pulp-like organoids using dental-pulp stem cells. *Cells*.

[11] Chu J., Pieles O., Pfeifer C. G., Alt V., Morsczeck C., Docheva D. (2021). Dental follicle cell differentiation towards periodontal ligament-like tissue in a self-assembly three-dimensional organoid model. *European Cells and Materials*.

[12] Abraham D. M., Herman C., Witek L., Cronstein B. N., Flores R. L., Coelho P. G. (2022). Self-assembling human skeletal organoids for disease modeling and drug testing. *Journal of Biomedical Materials Research. Part B, Applied Biomaterials*.

[13] Crispim J. F., Ito K. (2021). De novo neo-hyaline-cartilage from bovine organoids in viscoelastic hydrogels. *Acta Biomaterialia*.

[14] Dominici M., Le Blanc K., Mueller I. (2006). Minimal criteria for defining multipotent mesenchymal stromal cells. The International Society for Cellular Therapy position statement. *Cytotherapy*.

[15] Shoushrah S. H., Transfeld J. L., Tonk C. H. (2021). Sinking our teeth in getting dental stem cells to clinics for bone regeneration. *International Journal of Molecular Sciences*.

[16] Gronthos S., Mankani M., Brahim J., Robey P. G., Shi S. (2000). Postnatal human dental pulp stem cells (DPSCs) in vitro and in vivo. *Proceedings of the National Academy of Sciences of the United States of America*.

[17] Zhang Q., Shi S., Liu Y. (2009). Mesenchymal stem cells derived from human gingiva are capable of immunomodulatory functions and ameliorate inflammation-related tissue destruction in experimental colitis. *Journal of Immunology*.

[18] Seo B. M., Miura M., Gronthos S. (2004). Investigation of multipotent postnatal stem cells from human periodontal ligament. *Lancet*.

[19] Santamaría S., Sanchez N., Sanz M., Garcia-Sanz J. A. (2017). Comparison of periodontal ligament and gingiva-derived mesenchymal stem cells for regenerative therapies. *Clinical Oral Investigations*.

[20] Gonçalves L. F., Fernandes A. P., Cosme-Silva L. (2016). Effect of EDTA on TGF-*β*1 released from the dentin matrix and its influence on dental pulp stem cell migration. *Brazilian Oral Research*.

[21] Sun B. Y., Zhao B. X., Zhu J. Y., Sun Z. P., Shi Y. A., Huang F. (2018). Role of TGF-*β*1 expressed in bone marrow-derived mesenchymal stem cells in promoting bone formation in a rabbit femoral defect model. *International Journal of Molecular Medicine*.

[22] Niwa T., Yamakoshi Y., Yamazaki H. (2018). The dynamics of TGF-*β* in dental pulp, odontoblasts and dentin. *Scientific Reports*.

[23] Arany P. R., Cho A., Hunt T. D. (2014). Photoactivation of endogenous latent transforming growth factor-*β*1 directs dental stem cell differentiation for regeneration. *Science Translational Medicine*.

[24] Jin S. H., Lee J. E., Yun J. H., Kim I., Ko Y., Park J. B. (2015). Isolation and characterization of human mesenchymal stem cells from gingival connective tissue. *Journal of Periodontal Research*.

[25] Son J., Tae J. Y., Min S. K., Ko Y., Park J. B. (2020). Fibroblast growth factor-4 maintains cellular viability while enhancing osteogenic differentiation of stem cell spheroids in part by regulating RUNX2 and BGLAP expression. *Experimental and Therapeutic Medicine*.

[26] Min S. K., Kim M., Park J. B. (2021). Insulin-like growth factor 2-enhanced osteogenic differentiation of stem cell spheroids by regulation of Runx2 and Col1 expression. *Experimental and Therapeutic Medicine*.

[27] Lee H., Lee H., Na C. B., Park J. B. (2019). The effects of simvastatin on cellular viability, stemness and osteogenic differentiation using 3-dimensional cultures of stem cells and osteoblast-like cells. *Advances in Clinical and Experimental Medicine*.

[28] Sasaki J. I., Abe G. L., Li A., Matsumoto T., Imazato S. (2021). Large three-dimensional cell constructs for tissue engineering. *Science and Technology of Advanced Materials*.

[29] Vaquette C., Saifzadeh S., Farag A., Hutmacher D. W., Ivanovski S. (2019). Periodontal tissue engineering with a multiphasic construct and cell sheets. *Journal of Dental Research*.

[30] Song Y. M., Lee H. J., Min S. K. (2022). Effects of noni on cellular viability and osteogenic differentiation of gingiva-derived stem cells demonstrated by RNA sequencing and quantitative PCR. *Experimental and Therapeutic Medicine*.

[31] Lee J. H., Song Y. M., Min S. K. (2021). NELL-1 increased the osteogenic differentiation and mRNA expression of spheroids composed of stem cells. *Medicina (Kaunas)*.

[32] Kim D., Lee A. E., Xu Q., Zhang Q., le A. D. (2021). Gingiva-derived mesenchymal stem cells: potential application in tissue engineering and regenerative medicine - a comprehensive review. *Frontiers in Immunology*.

[33] Lee H., Lee S. I., Ko Y., Park J. B. (2018). Evaluation of the secretion and release of vascular endothelial growth factor from two-dimensional culture and three-dimensional cell spheroids formed with stem cells and osteoprecursor cells. *Advances in Clinical and Experimental Medicine*.

[34] Kim B. B., Tae J. Y., Ko Y., Park J. B. (2019). Lovastatin increases the proliferation and osteoblastic differentiation of human gingiva-derived stem cells in three-dimensional cultures. *Experimental and Therapeutic Medicine*.

[35] Tam W. L., Mendes L. F., Chen X. (2021). Human pluripotent stem cell-derived cartilaginous organoids promote scaffold-free healing of critical size long bone defects. *Stem Cell Research & Therapy*.

[36] Kang S. H., Park J. B., Kim I., Lee W., Kim H. (2019). Assessment of stem cell viability in the initial healing period in rabbits with a cranial bone defect according to the type and form of scaffold. *Journal of Periodontal & Implant Science*.

[37] Pittenger M. F., Discher D. E., Péault B. M., Phinney D. G., Hare J. M., Caplan A. I. (2019). Mesenchymal stem cell perspective: cell biology to clinical progress. *NPJ Regenerative Medicine*.

[38] Yin X., Mead B. E., Safaee H., Langer R., Karp J. M., Levy O. (2016). Engineering stem cell organoids. *Cell Stem Cell*.

[39] Scalise M., Marino F., Salerno L. (2021). From spheroids to organoids: the next generation of model systems of human cardiac regeneration in a dish. *International Journal of Molecular Sciences*.

[40] Lou Y. R., Leung A. W. (2018). Next generation organoids for biomedical research and applications. *Biotechnology Advances*.

[41] Li H., Gao L., du J., Ma T., Ye Z., Li Z. (2021). To better generate organoids, what can we learn from teratomas?. *Frontiers in Cell and Developmental Biology*.

[42] Ng J., Wei Y., Zhou B., Burapachaisri A., Guo E., Vunjak-Novakovic G. (2016). Extracellular matrix components and culture regimen selectively regulate cartilage formation by self-assembling human mesenchymal stem cells in vitro and in vivo. *Stem Cell Research & Therapy*.

[43] Iwata J., Hosokawa R., Sanchez-Lara P. A., Urata M., Slavkin H., Chai Y. (2010). Transforming growth factor-beta regulates basal transcriptional regulatory machinery to control cell proliferation and differentiation in cranial neural crest-derived osteoprogenitor cells. *The Journal of Biological Chemistry*.

[44] Ahn Y. H., Kim T. H., Choi H. (2015). Disruption of Tgfbr2 in odontoblasts leads to aberrant pulp calcification. *Journal of Dental Research*.

[45] Sloan A. J., Couble M. L., Bleicher F., Magloire H., Smith A. J., Farges J. C. (2001). Expression of TGF-*β* receptors I and II in the human dental pulp by in situ hybridization. *Advances in Dental Research*.

[46] Sloan A. J., Matthews J. B., Smith A. J. (1999). TGF-beta receptor expression in human odontoblasts and pulpal cells. *The Histochemical Journal*.

[47] Hwang Y. C., Hwang I. N., Oh W. M., Park J. C., Lee D. S., Son H. H. (2008). Influence of TGF-beta1 on the expression of BSP, DSP, TGF-beta1 receptor I and Smad proteins during reparative dentinogenesis. *Journal of Molecular Histology*.

[48] Wang Y., Cox M. K., Coricor G., MacDougall M., Serra R. (2013). Inactivation of Tgfbr2 in Osterix-Cre expressing dental mesenchyme disrupts molar root formation. *Developmental Biology*.

[49] Zhang R., Lin J., Liu Y. (2021). Transforming growth factor-*β* signaling regulates tooth root dentinogenesis by cooperation with Wnt signaling. *Frontiers in Cell and Developmental Biology*.

[50] O'Connor S. K., Katz D. B., Oswald S. J., Groneck L., Guilak F. (2021). Formation of osteochondral organoids from murine induced pluripotent stem cells. *Tissue Engineering. Part A*.

[51] Fujiike A. Y., Lee C. Y. A. L., Rodrigues F. S. T. (2022). Anticancer effects of carboxymethylated (1→3)(1→6)-*β*-D-glucan (botryosphaeran) on multicellular tumor spheroids of MCF-7 cells as a model of breast cancer. *Journal of Toxicology and Environmental Health, Part A*.

[52] Park J. B., Zhang H., Lin C. Y. (2012). Simvastatin maintains osteoblastic viability while promoting differentiation by partially regulating the expressions of estrogen receptors *α*. *The Journal of Surgical Research*.

[53] Lee H. J., Song Y. M., Baek S., Park Y. H., Park J. B. (2021). Vitamin D enhanced the osteogenic differentiation of cell spheroids composed of bone marrow stem cells. *Medicina (Kaunas)*.

[54] Chen S., Gluhak-Heinrich J., Wang Y. H. (2009). Runx2, osx, and dspp in tooth development. *Journal of Dental Research*.

[55] Wu J., Li N., Fan Y. (2019). The conditioned medium of calcined tooth powder promotes the osteogenic and odontogenic differentiation of human dental pulp stem cells via MAPK signaling pathways. *Stem Cells International*.

[56] Kawasaki K., Kawasaki M., Watanabe M. (2015). Expression of Sox genes in tooth development. *The International Journal of Developmental Biology*.

[57] Mina M., Braut A. (2004). New insight into progenitor/stem cells in dental pulp using Col1a1-GFP transgenes. *Cells, Tissues, Organs*.

